# Biphasic Catalytic Conversion of Olefins in Aqueous Media: A Systematic Review

**DOI:** 10.3390/ijms26094028

**Published:** 2025-04-24

**Authors:** Angeliki Chira, Nikolaos C. Kokkinos

**Affiliations:** 1 Department of Chemistry, School of Sciences, Democritus University of Thrace, Ag. Loukas, 654 04 Kavala, Greece; 2 Petroleum Institute, Democritus University of Thrace, Ag. Loukas, 654 04 Kavala, Greece; 3 Hephaestus Laboratory, School of Sciences, Democritus University of Thrace, Ag. Loukas, 654 04 Kavala, Greece

**Keywords:** aqueous biphasic catalysis, olefins, heterogenized homogeneous catalysts, Wacker oxidation, hydrogenation, hydroformylation, epoxidation, hydro aminomethylation

## Abstract

Aqueous biphasic catalysis has gained recognition as a sustainable and efficient method that combines the advantages of both homogeneous and heterogeneous catalytic systems. This approach enables the separation and recycling of catalysts, leading to reduced environmental impact and lower operational costs. A key component of this method is the use of transition metal catalysts, which are crucial for facilitating various reactions when paired with different types of ligands, primarily hydrophiles. This combination is essential for achieving high success rates in recyclable catalytic systems. The reaction conditions, including temperature, pressure, and pH, significantly influence catalytic performance. However, challenges such as limited substrate solubility and catalyst leaching persist, underscoring the need for further research into advanced ligand design, catalyst immobilization techniques, and scalable process integration. This review systematically examines recent experiments in the aqueous biphasic catalysis of olefins, following the Preferred Reporting Items for Systematic Reviews and Meta-Analyses framework. From an initial pool of 597 articles, 104 were found to be relevant and focused specifically on aqueous biphasic catalysis. The study investigates key reactions, the factors that influence these biphasic reactions, and the catalytic systems that facilitate them. By highlighting both progress and ongoing challenges, this work underscores the potential of aqueous biphasic catalysis to bridge the gap between green chemistry principles and industrial applications.

## 1. Introduction

As the economic crisis worsens and climate change continues to pose challenges, researchers are constantly exploring new methods for conducting catalytic reactions. The goal is to achieve the most effective and efficient use of energy while minimizing harm to the environment. Aqueous biphasic catalysis presents an innovative method for conducting chemical reactions by combining the advantages of both homogeneous and heterogeneous catalysis. Homogeneous catalysis is known for its high selectivity and ability to operate under mild conditions, while heterogeneous catalysis facilitates the easy separation of the catalyst from the product. In biphasic catalysis, the catalyst is immobilized in a separate liquid phase that does not mix with the products, enabling the efficient recycling of homogeneous catalysts. Once the reaction reaches the desired stage, stirring is stopped, and the mixture separates into two distinct layers: one containing the product and the other containing the catalyst. This separation can be accomplished through simple decantation, allowing for the immediate reuse of the catalyst solution [1]. The concept of two-phase catalysis, particularly in aqueous systems, experienced significant development in the 1980s, especially with the introduction of two-phase hydroformylation by Ruhrchemie and Rhône-Poulenc (RCH/RP). This technique, first applied to the industrial production of n-butyraldehyde in 1984, has since become one of the most important methods in modern organic chemistry [2]. Water is widely recognized as the most environmentally friendly solvent for biphasic catalysis, primarily due to its economic advantages, ecological benefits, and safety [3]. Its use aligns with the principles of green chemistry, as it helps minimize the environmental impact of chemical processes. However, the low solubility of organic reactants, particularly higher olefins, poses significant limitations on the industrial application of aqueous biphasic hydroformylation and other reactions, restricting its use primarily to short-chain olefins [1]. In this aqueous biphasic reaction system, the catalyst is immobilized relative to the substrates, while the catalytic process itself remains homogeneous. The activity of the catalyst is influenced by mass transfer phenomena, which led to the development of various strategies to modify interfacial properties and address the mass transfer limitations encountered with long-chain olefins [4]. These strategies include the incorporation of co-solvents or surfactants to enhance solubility, the use of thermo-regulated phase-transfer catalysts to facilitate reactant transfer between phases, the application of supported aqueous-phase catalysts (SAPC) to disperse an aqueous catalyst solution onto a solid support, and the integration of amphiphilic ligands to improve the interactions between catalysts and olefins in an aqueous environment [4,5]. These approaches aim to improve reaction rates and overall efficiency, making aqueous biphasic reactions more viable for a broader range of olefins.

Two-phase catalysis is influenced by a multitude of factors, which determine the course and efficiency of the reaction. Among these, the conditions under which the reaction takes place, such as the temperature, pressure, stirring rate and pH, play a crucial role, while the presence of additional components can significantly affect the kinetics and selectivity of the process. In particular, elements such as electrolytes, polymers, nanoparticles, cyclodextrins and other specialized additives have been intensively investigated, with the aim of improving the solubility, stability and performance of the catalytic system, as reported in this study. The central factor that determines the success of two-phase catalysis is the catalytic system. In this context, rhodium complexes have dominated the field of aqueous two-phase catalysis due to their high activity and selectivity. However, research is not limited to them, as new catalysts and solvents are continuously being developed and applied, aiming to optimize the performance, stability, and environmental sustainability of the process. The use of alternative solvents, the synthesis of new catalytic structures and the incorporation of innovative additives are active areas of research aiming to address the challenges encountered in the scalable application of these technologies [6,7].

Despite significant advances, challenges remain in the aqueous biphasic catalysis of olefins. These include minimizing catalyst leaching from aqueous to organic phases for efficient recycling; overcoming the mass transfer limitations for long-chain olefins; and developing methods to control and optimize the interfacial area for maximum catalytic activity [5,8]. Future research opportunities include developing novel ligands that enhance the solubility and reactivity of olefins in water; optimizing reactor designs to improve mixing and mass transfer; and applying advanced analytical techniques to better understand interfacial phenomena and reaction mechanisms. The aqueous biphasic catalysis of olefins represents a promising research area with significant potential for industrial applications. By addressing the challenges related to substrate solubility, mass transfer limitations, and catalyst leaching, it is possible to develop highly efficient and environmentally friendly catalytic processes. This study aims to explore recent advances in aqueous biphasic catalysis, focusing on catalytic systems and factors influencing the overall reactions. Through a comprehensive analysis of these aspects, this work seeks to provide insights into designing and optimizing aqueous biphasic catalytic systems for olefin hydroformylation and related reactions.

## 2. Methodology

**Figure 1:**
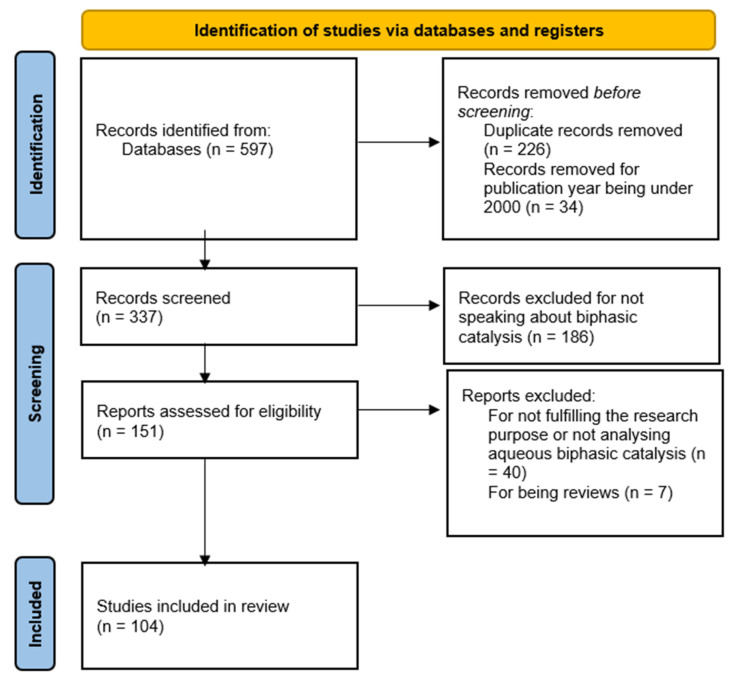
Preferred Reporting Items for Systematic Reviews and Meta-Analyses (PRISMA) flowchart of the systematic review study.

The aqueous biphasic catalysis of olefins is a rapidly growing research field. This analysis aims to systematically explore the relevant literature, following a methodical approach to identify, select, and analyze the most relevant research papers. This systematic review was conducted using the Preferred Reporting Items for Systematic Reviews and Meta-Analyses (PRISMA), as shown in Figure 1. Scopus, Google Scholar, and ScienceDirect were used to search for the most relevant literature to this review’s topic. The methodology employed in this analysis proved to be the most effective. Initially, a preliminary search indicated the existence of hundreds of articles related to the aqueous two-phase catalysis of olefins. However, upon further examination, it became apparent that the actual number of relevant articles was significantly lower than initially perceived. The database search was conducted from 16 October 2024 to 23 November 2024 for article titles, abstracts, and keywords with publication dates between 2000 and 2025. The search was initiated on Scopus using the keywords ‘biphasic AND catalysis’, including the keywords ‘aqueous’ and ‘olefins OR alkenes’ in the advanced search section. To continue the search without any useful articles being lost, the following terms were searched on all databases: ‘aqueous biphasic catalytic conversion of olefins’, ‘aqueous biphasic catalytic conversion of alkenes’, ‘biphasic homogeneous catalysis of olefins’, ‘biphasic homogeneous catalysis of alkenes’, ‘aqueous biphasic hydroformylation of olefins’, ‘aqueous biphasic hydroformylation of alkenes’, ‘aqueous biphasic hydrogenation of olefins’, ‘aqueous biphasic hydrogenation of alkenes’, ‘olefins in aqueous biphasic catalytic systems’, ‘alkenes in aqueous biphasic catalytic systems’, ‘heterogenized homogeneous catalysis of olefins’, ‘heterogenized homogeneous catalysis of alkenes’, ‘aqueous biphasic polymerization of olefins’, ‘aqueous biphasic polymerization of alkenes’, ‘aqueous biphasic alkylation of olefins’, ‘aqueous biphasic alkylation of alkenes’, ‘aqueous biphasic epoxidation of olefins’, ‘aqueous biphasic epoxidation of alkenes’, ‘aqueous biphasic oligomerization of olefins’, ‘aqueous biphasic oligomerization of alkenes’, ‘aqueous biphasic hydroamination of olefins’, ‘aqueous biphasic hydroamination of alkenes’, ‘aqueous biphasic isomerization of olefins’, ‘aqueous biphasic isomerization of alkenes’, ‘aqueous biphasic ozonolysis of olefins’, ‘aqueous biphasic ozonolysis of alkenes’, ‘heterogenization of homogeneous catalysis of olefins’, ‘heterogenization of homogeneous catalysis of alkenes’, ‘aqueous organometallic catalysis of olefins’, and ‘aqueous organometallic catalysis of alkenes’. In summary, 597 relevant articles were found and exported to a spreadsheet, including the Title, the Abstract, and the Year of Publication. Duplicates (226) were found and removed. Moreover, 34 articles were removed before screening due to the year of publication being under 2000. As the screening started, a cursory review of each abstract and title was performed. An additional 186 articles were removed because they were not compliant with the research criteria, indicating that they did not pertain to biphasic catalysis, but rather focused on either homogeneous or heterogeneous catalysis. Following another thorough analysis, a total of 104 papers were incorporated into this study. However, 40 records were excluded due to their primary focus being on biphasic catalysis involving alternative solvents rather than aqueous biphasic catalysis. Furthermore, seven records were removed as they were review articles that covered experiments discussed in earlier studies.

## 3. Results and Discussion

The technique of biphasic catalytic reactions is a highly versatile and efficient methodology that is applicable to a wide range of chemical transformations. This approach is designed not only to enhance the overall efficiency and selectivity of reactions but also to offer significant economic and practical advantages, particularly in terms of catalyst recovery and recycling. One of the major limitations of traditional homogeneous catalysis lies in the difficulty of separating and reusing the catalyst, leading to increased costs and potential environmental concerns. Biphasic catalysis, also known as heterogenized homogeneous catalysis, addresses this issue by combining the superior reactivity and selectivity of homogeneous systems with the cost-effectiveness and sustainability of heterogeneous catalysis. Extensive studies have demonstrated that the use of biphasic catalysis can lead to significantly improved conversion rates and product yields across various reactions, further highlighting its potential as a highly efficient catalytic strategy. By leveraging the advantages of both catalytic systems, this method not only enhances the reaction performance but also contributes to greener and more economically viable industrial processes by reducing waste generation and minimizing catalyst losses. In the following sections, the presented tables systematically categorize different reactions, olefin groups, and their respective outcomes, offering a structured approach to analyzing reaction performance under biphasic catalytic conditions. This classification enables a more comprehensive discussion of the catalytic efficiency achieved in each case and provides valuable insights into the extent to which biphasic catalysis serves as a truly effective and scalable approach to various chemical processes.

**Figure 2:**
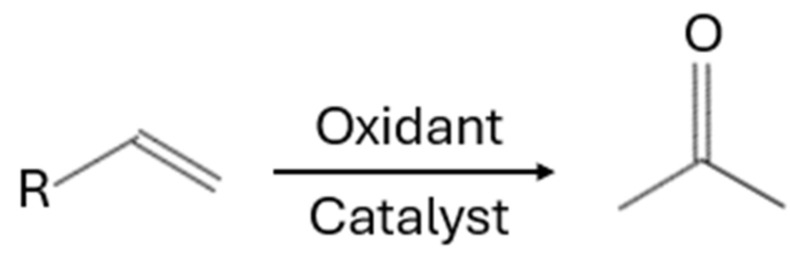
Wacker oxidation reaction.

The Wacker oxidation reaction (Figure 2) is a commonly employed method for oxidizing olefins into carbonyl compounds, such as ketones. The reaction involves the catalytic complex, which activates the alkene toward nucleophilic attack by water. The resulting intermediate is then oxidized to a carbonyl compound, and the catalyst is usually regenerated by a co-oxidant, allowing the cycle to continue.

As illustrated in Table 1, the conversion rates for both larger olefins, including C_12_–C_20_, and smaller olefins are notably high, often surpassing 90%. In certain cases, these rates can even reach as high as 99%, accompanied by a selectivity of 99%. The selection of an appropriate catalyst is crucial, as it must exhibit sufficient selectivity to yield the desired products, such as ketones. During Wacker oxidation, various side reactions can occur due to the harsh conditions and the formation of active intermediates. One significant undesired reaction is the peroxidation of the product; the produced ketone can be further oxidized to a carboxylic acid. Additionally, the presence of chlorides and metal catalysts can result in the chlorination of the alkene, leading to the generation of chlorinated by-products through electrophilic additions. Another problematic process is the isomerization of the double bond, which may produce different carbonyl compounds than anticipated. Furthermore, alkenes can participate in coupling reactions that result in the formation of dimers or oligomers [9,10]. In cyclic compounds such as cyclohexane and styrene, the yield appears to be limited to approximately 39%. This constraint may arise from the fact that chitosan is not ideally suited to this reaction, as it exhibits relative instability in acidic environments. Nevertheless, the reaction leads to noteworthy results and observations. For example, immobilized catalysts show enhanced activity and selectivity compared to homogeneous catalysts, with Turn-Over Frequency (TOF) values ranging from 12 to 160 h^−1^ [11]. TOF refers to the number of substrate molecules that a single catalytic site can convert per hour, serving as a key metric for comparing catalytic efficiency under specific conditions. Palladium (Pd) and manganese (Mn) are pivotal to the catalysts used for this reaction. Both metals excel as catalysts due to their high selectivity, stability, and relatively low cost, particularly manganese, which is abundant and well-suited for green chemistry applications, including aqueous biphasic catalysis. A variety of oxidizing agents were employed in different amounts, but none emerged as particularly noteworthy. The reaction usually occurs under mild pressure and temperature conditions, primarily within a temperature range of 25 to 100 degrees Celsius. This range is not considered particularly high or cost-prohibitive, making it ideal for producing the main product while minimizing the formation of by-products. The duration of the experiments varies from 2 to 24 h, a range that is standard for such procedures. This extended duration is particularly important at lower temperatures, where the additional time allows for optimal interaction between the reactants and the catalyst at the solvent’s interface. To support this interaction, agitation is conducted at a consistent rate of 300 rpm [9]. Finally, several additives were examined during the processes, including paraffins, which not only reduced isomerization but also enhanced overall selectivity, and NaOAc and NaOH compounds, which contributed to a highly selective and recyclable catalytic system capable of enduring multiple catalytic cycles. This characteristic is a common advantage in this type of reaction, as two-phase catalysis allows for the reuse of the catalyst.

**Figure 3:**
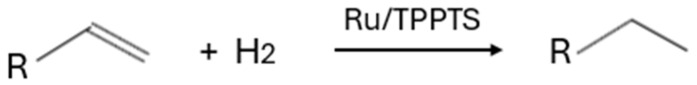
Hydrogenation reaction of olefins.

Beyond oxidation reactions, the hydrogenation of olefins (Figure 3) represents another fundamental transformation in both industrial and academic chemistry, with significant applications in fuel processing and fine chemical synthesis. The aqueous two-phase hydrogenation method aims to enhance the recyclability of the catalyst while preserving high levels of conversion and selectivity. In this process, hydrogen gas is introduced and the catalyst facilitates the addition of hydrogen atoms across the double bond of the olefin. After the reaction, the catalyst remains in the aqueous layer, enabling its easy separation and reuse.

As demonstrated in Table 2, a variety of rhodium and ruthenium complexes [12,13,14,15,16,17,18,19,20,21,22,23], palladium and nickel nanoparticles, and Raney Ni [24,25,26,27,28] and cobalt complexes [29] are utilized as catalysts in this process. Rhodium complexes are recognized for their high activity and selectivity, establishing them as a prominent choice in catalyst production. The incorporation of water-soluble phosphine substituents, such as TPPMS and TPPTS, significantly enhances the solubility of the catalyst in aqueous environments, thereby facilitating its efficient recovery [13,14]. Ruthenium and palladium catalysts embedded in polymeric microspheres enhance activity while minimizing unwanted reactions due to the microenvironment they create [26,28]. Cobalt complexes demonstrate excellent selectivity and can achieve conversions of up to 100%. In contrast, Raney Ni, while more economical, shows lower selectivity and is more susceptible to over-hydrogenation and isomerization [24]. Notably, the catalyst HRh(CO)(TPPMS)_3_ exhibited remarkable resistance to sulfide compounds, making it suitable for use in systems with higher sulfur content, such as naphtha fractions [12]. In recent years, the use of nanoparticles as catalysts has also attracted particular interest as they combine high activity with innovative capabilities. In particular, metal nanoparticles such as ruthenium (Ru), rhodium (Rh), and platinum (Pt) exhibit excellent selectivity, stability, and reusability, making them ideal for catalytic hydrogenation. Ruthenium nanoparticles achieve the selective hydrogenation of alfa-pinene, combining homogeneous behavior with ease of recovery. Rhodium nanoparticles, thanks to the use of thermoregulated ligands, allows for smart phase transfer and easy recycling, while the use of platinum nanoparticles in continuous flow systems offers increased efficiency and suitable conditions for the industrial scale. In all three cases, the high specific surface area of the nanoparticles leads to increased activity, while the reusability contributes to the sustainability of the processes [20,21,22]. The reaction conditions can vary significantly, with temperatures ranging from 25 °C to 150 °C, hydrogen pressures varying between 0.99 atm and 98.7 atm, and reaction times spanning from 1 to 24 h. Lower temperatures (20–60 °C) tend to favor the selectivity of partially hydrogenated products, such as alcohols, especially when hydroformylated olefins are used as feedstock [25,26,27], while higher temperatures, above 100 °C, result in the complete hydrogenation of compounds to form alkanes [12,13,14]. Increasing hydrogen pressure speeds up the reaction, which enhances both the conversion and yield. However, it may also decrease selectivity [12,13,15]. Reaction times exceeding 3 to 10 h can result in undesirable side effects, such as isomerization or oligomerization, particularly when using non-noble metal catalysts. These extended reaction times generally decrease both conversion rates and yield. This is likely because branched olefins tend to be less reactive than linear ones [15,27]. Furthermore, high stirring speeds of 1600 to 2000 rpm enhance mass transfer and increase reaction efficiency [24], although speeds as low as 600 rpm under appropriate conditions can also yield optimal results depending on the catalyst present [12]. The addition of electrolytes such as NaCl or Na_2_SO_4_ enhances the reaction by increasing the coordination of aldehydes to the catalyst, while potassium salts (e.g., KCl, K_2_SO_4_) decrease the yield [27]. Surfactants like CTAB and Brij-35 enhance substrate solubility and catalyst dispersion [23]. The recyclability of catalysts is critically important. Studies have shown that there is minimal loss of rhodium to the organic phase, even after many hours of operation. In particular, rhodium catalysts with thermoregulating substituents demonstrate remarkable durability, maintaining over 87% conversion even after multiple recycling cycles [20]. Lastly, the Turnover Frequency (TOF) number is an important parameter for effectively characterizing a reaction and its catalyst. Using simple rhodium complexes, turnover frequency (TOF) numbers of 255–2000 h^−1^ were achieved. The use of nanoparticles as catalysts resulted in TOF numbers ranging from 500 to 2000 h^−1^ under relatively mild conditions, highlighting their significance in the field of biphasic catalysis. In contrast, simple Rh/TPPTS complexes achieved notable TOF numbers between 255 and 1245 h^−1^ under moderate conditions, particularly with the addition of cationic DTAC and Brij-35 [20,23]. Despite its impressive results, aqueous two-phase hydrogenation still faces several challenges. These include the limited range of substrates, particularly for larger or branched molecules, the need to optimize phase transfer conditions, and the necessity of scaling up the process for industrial applications. A promising approach involves using rhodium complexes with phosphine substituents, along with stabilizers such as electrolytes and surfactants. Future research will focus on expanding the range of applicable substrates, enhancing the stability of the catalysts, and increasing yields under industrial conditions.

**Figure 4:**
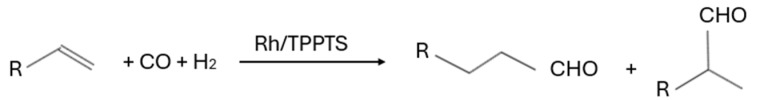
Hydroformylation reaction.

Building upon these catalytic advancements, aqueous biphasic hydroformylation further enhances olefin conversion by enabling selective aldehyde formation under optimized reaction conditions. Aqueous biphasic hydroformylation is a crucial reaction in organometallic catalysis, enabling the conversion of olefins to aldehydes with high selectivity and efficiency. Τhe hydroformylation reaction (Figure 4) involves the addition of a formyl group and a hydrogen atom across the carbon–carbon double bond of an olefin using synthesis gas (a mixture of CO and H_2_). The catalyst, typically a transition metal complex, facilitates the activation of the olefin and the coordination and insertion of CO and H_2_, driving the formation of the aldehyde product.

As shown in Table 3, this method is based on the use of catalysts containing transition metals such as rhodium (Rh) [1,3,6,30,31,32,33,34,35,36,37,38,39,40,41,42,43,44,45,46,47,48,49,50,51,52,53,54,55,56,57,58,59,60,61,62,63,64,65,66,67,68,69,70,71,72,73,74,75,76,77,78,79,80,81,82,83,84,85,86,87,88] and cobalt (Co) [89,90,91], in combination with water-soluble phosphines such as TPPTS and other ligands such as acetylacetones (acac), which optimize the efficiency and selectivity of the reaction. Rhodium is predominantly used in this reaction, and its extensive experimental applications confirm its status as one of the best metals for catalysts due to its exceptional properties. Common complexes such as RhH(CO)(TPPTS)_3_ and RhCl(CO)(TPPTS)_2_ demonstrate high activity and stability in aqueous environments [31,32,34,36,38,40,41,45,46,67]. While cobalt catalysts like CoCl_2_(TPPTS)_2_ can yield acceptable results across various substrates, rhodium typically demonstrates greater activity [91,92]. The addition of water-soluble phosphine ligands, like TPPTS (trisodium triphenylphosphine trisulfonate), enhances the catalyst’s solubility in the water–organic two-phase system, making recovery and recycling easier [53,54]. TPPTS has been shown to enhance the selectivity for linear aldehydes. Meanwhile, the addition of modified phosphines, such as TPPDS and CDPPDS, influences both the reaction kinetics and the ratio of linear to branched aldehydes [45,46,47]. The use of multifunctional phosphines, like TPPTS-modified SiO_2_, enhances catalyst stability and selectivity [48]. While other ligands also exhibit excellent catalytic abilities, phosphine catalysts have been the preferred choice for many decades. This preference is largely due to their high water solubility, which is achieved through the use of sulfonates. This property is particularly significant in the field of green chemistry, where the main goal is either to avoid using solvents altogether or to utilize water as a solvent. Additionally, phosphine catalysts possess unique characteristics that enhance their activity and selectivity. In recent years, Sulfoxantphos has emerged as a particularly important bidentate phosphine ligand, with widespread use in hydroformylation reactions as well as in other transformations in biphasic aqueous systems, especially when β-cyclodextrins (RAME-β-CD) are present, though not exclusively. It stands out for its excellent regioselectivity (l/b ratio), reaching values as high as 32–34 in the hydroformylation of long-chain alkenes such as 1-decene. This is attributed to the creation of a sterically compact environment around the metal center, which promotes linear-selective CO insertion. In contrast to other water-soluble phosphine ligands (such as TPPTS), Sulfoxantphos exhibits minimal interaction with cyclodextrins and does not form stable complexes, thus avoiding catalyst deactivation. Furthermore, catalytic systems containing Sulfoxantphos are stable and recyclable, with metal or phosphorus losses below 0.003% h^−1^ during continuous operation, reflecting the strong binding of the ligand to the metal center. The versatility of Sulfoxantphos makes it suitable for hydrophobic substrates, such as 1-hexadecene, offering good selectivity and performance. At the same time, its high compatibility with continuous processes, as demonstrated in a miniplant setup with productivity up to 1.5 kg of product per mg of lost Rh, underscores its industrial relevance. However, a key drawback of these systems is their relatively lower catalytic activity, necessitating elevated temperatures (120–125 °C) to achieve satisfactory reaction rates, particularly when compared to other systems. Overall, Sulfoxantphos is an ideal choice for biphasic catalytic applications, combining a high selectivity, recyclability, chemical stability, and low levels of interaction with cyclodextrins, making it especially attractive for sustainable, industrial-scale processes [93,94]. The conditions of the reaction also play a crucial role in determining the efficiency of the process. Typically, the temperature ranges from 70 to 130 °C, while the pressure of the synthesis gas (syngas, a mixture of CO and H_2_) varies between 9.9 and 98.7 atm. Controlling the temperature and pressure is essential for regulating the selectivity of the reaction. At higher temperatures, the tendency toward olefin isomerization increases, which can lead to a decrease in selectivity towards linear aldehydes. Additionally, elevated temperatures can promote side reactions such as further aldehyde reduction to alcohols, aldol condensation, and even catalyst decomposition or ligand degradation, especially in cobalt- or rhodium-based systems. These effects collectively reduce the overall yield and selectivity in hydroformylation processes. Additionally, at elevated temperatures, the catalyst may transition to the organic phase, which is unwanted since the reaction takes place in the interface [61]. Moreover, the CO:H_2_ ratio influences product distribution, with higher CO concentrations favoring the formation of oxygenated derivatives [34]. The use of organic solvents, such as polyethylene glycols, can improve the solubility of propene in water, thereby accelerating the overall process [30]. Additives are crucial for enhancing the catalytic system’s activity. Ionic liquids like [OctMim]Br boost substrate solubility in the two-phase matrix, leading to improved mass transfer and reaction rates [49]. Furthermore, cyclodextrins (RAME-β-CD) create inclusion complexes with hydrophobic olefins, enhancing the separability of the organic and aqueous phases while facilitating catalyst recycling [51,54,60,70,84]. The use of non-ionic and cationic surfactants, such as Brij-35 and SDS, has been shown to enhance the efficiency of hydroformylation. This is achieved through the formation of micelle structures that improve the interaction between the catalyst and the substrate. Additionally, hydrogen bonds between water and multifunctional phosphines contribute to the stability of the catalyst by reducing the likelihood of deactivation due to complex degradation [87]. The catalytic activity of these systems is characterized by high yields and high selectivity. The conversion rates range from 75% to 100%, while the selectivity for aldehydes varies between 40% and 99.5%, depending on the specific conditions and type of catalyst used. The turnover frequency (TOF) can reach up to 5046 h^−1^ with certain surfactants, and even without any additives, it can achieve up to 1614 h^−1^, demonstrating the excellent efficiency of the systems employed [48,67]. Conversely, the Turnover Number (TON), defined as the number of product molecules generated per molecule of catalyst prior to deactivation, varies between 8.95 and 365. The highest recorded value belongs to a Cobalt (Co)-based catalyst, demonstrating the exceptional efficiency of this system regarding its catalytic activity [61]. In certain instances, the stability of the catalyst is remarkable, allowing it to maintain its performance for h without a significant loss of activity. This demonstrates the viability of the approach for industrial applications. In conclusion, aqueous two-phase hydroformylation is an efficient method for converting olefins to aldehydes, offering notable advantages in catalyst recycling and a reduced environmental impact. The continuous optimization of catalyst systems, along with the development of new additives and ligands, aims to enhance efficiency and selectivity, paving the way for more sustainable industrial applications.

**Figure 5:**
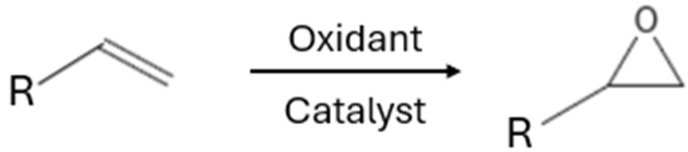
Epoxidation reaction.

In addition to hydroformylation, the epoxidation of olefins (Figure 5) plays a crucial role in organic and industrial chemistry, as epoxides serve as key intermediates in the synthesis of pharmaceuticals and fine chemicals. The epoxidation reaction of olefins is a vital process in both organic and industrial chemistry, as epoxides serve as important intermediates for the synthesis of pharmaceuticals, polymers, and fine chemicals. This reaction involves the oxidative conversion of an alkene’s double bond into a three-membered epoxide ring, facilitated by an oxidizing agent and a catalyst. Notably, the aqueous biphasic epoxidation of olefins enables the optimization of the reaction from both environmental and economic perspectives, as it eliminates the need for toxic and non-recyclable solvents.

Epoxidation is conducted using various catalytic systems, as shown in Table 4. Polyoxometallic compounds (POMs) and dimethyldioxirane (DMDO), which is formed in situ from oxone and acetone, play a crucial role in this process. Additionally, metal porphyrins and various oxidizing agents, such as hydrogen peroxide (H_2_O_2_) and oxone (potassium peroxymonosulfate), are also used [96,97,98,99,100]. These catalysts enable efficient and selective reactions under mild temperature conditions (25–70 °C) and within short reaction times (from a few min to 4 h). The use of H_2_O_2_ as an oxidizing agent allows for a high conversion rate of olefins and a selectivity of up to 99.5%. This is due to the purity of the system and the absence of unwanted by-products [96,97,98,99]. In the case of polyoxometallic derivatives, the reaction temperature is 70 °C, with an efficiency up to 95% [99]. The terpene epoxidation system achieves excellent selectivity, reaching 100% conversion and efficiency. This is a significant finding, as terpene epoxides are crucial in the production of biomaterials and in the pharmaceutical industry [100]. The use of specialized additives like hexafluoroacetone hydrate (HFAH) enhances the selectivity and stability of the catalytic system by reducing side reactions, such as epoxide ring-opening (via hydrolysis, alcoholysis, or acid/base-catalyzed rearrangements), overoxidation (e.g., Baeyer–Villiger oxidation or oxidative cleavage), and competing pathways like allylic oxidation or isomerization to carbonyl compounds. These additives help maintain the integrity of the epoxide product and minimize catalyst deactivation [98]. Overall, biphasic conditions, the use of polyoxometallic catalysts, and the selection of a suitable oxidant make epoxidation efficient and environmentally friendly, enhancing its potential for industrial applications.

**Figure 6:**
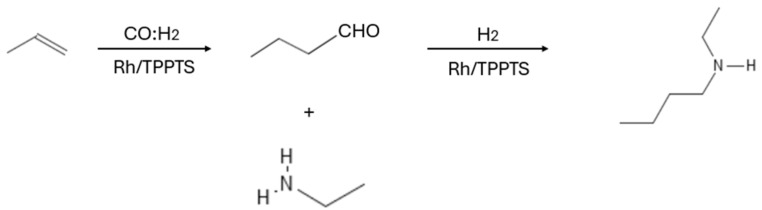
Hydroaminomethylation reaction of olefins.

Expanding on the applications of aqueous biphasic catalysis, the hydroaminomethylation of olefins (Figure 6) provides an efficient route for the selective synthesis of amines, which are vital compounds in numerous industrial processes. The aqueous biphasic hydroaminomethylation of olefins is a significant reaction for synthesizing amines with high selectivity. This reaction involves the formation of an intermediate aldehyde via hydroformylation, followed by reductive amination with an amine source. The catalyst plays a key role in both steps, first promoting the addition of CO and H_2_ to the olefin to form the aldehyde and then facilitating its subsequent conversion to the desired amine under hydrogenation conditions.

In Table 5, the reaction was conducted using rhodium (Rh)-based catalytic systems. These include the Rh/TPPTS catalyst, [Rh(cod)Cl] combined with sulfated phosphines (Sulfoxantphos), and [Rh(acac)(CO)_2_] [93,94,101,102,103,104,105]. These catalytic systems, when combined with the right temperature, pressure, and additives, make the process highly efficient. The reaction occurs at high temperatures ranging from 80 to 130 °C, with pressure varying between 29.6 and 59.2 atm, depending on the substrate used. For small olefins (C_2_–C_12_), the use of two-phase catalytic systems that include Rh nanoparticles and phosphines, along with the addition of morpholinates and CTAB, achieves conversion efficiencies of nearly 99%. The selectivity in this process ranges from 51.4% to 98% [102,103,105]. CTAB plays a crucial role in speeding up the reaction by enhancing the interfacial area and increasing the concentration of rhodium on the surface. However, higher temperatures can reduce the selectivity towards linear amines due to the occurrence of favorable side reactions such as the over-hydrogenation of intermediates, aldol condensation of aldehydes, isomerization or polymerization of imines/enamines, and the formation of regioisomeric mixtures, especially with internal alkenes. On the other hand, while increasing the pressure improves the overall conversion, it tends to compromise the selectivity [103]. For larger olefins (C_10_–C_16_), using Rh/TPPTS with Rh(acac)(CO)_2_ and Sulfoxantphos, combined with the RAME-β-CD additive, achieves 80% selectivity [93,94]. The use of the cyclodextrin agent RAME-β-CD likely enhances the solubility and stability of the catalytic system. In summary, choosing the right catalyst, conditions, and additives can significantly influence the performance and selectivity of hydroaminomethylation. This makes the reaction especially vital for the industrial production of specialized amines.

**Figure 7:**
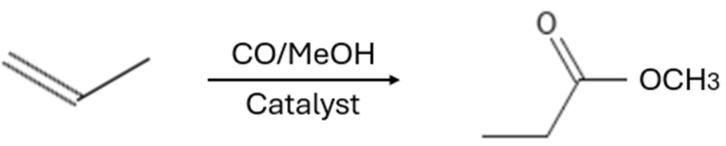
Methoxycarbonylation reaction of olefins.

**Figure 8:**
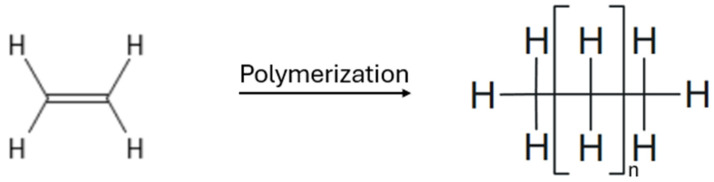
Polymerization reaction of olefins.

Beyond these key transformations, aqueous two-phase catalysis has been employed in a variety of other organic reactions, including cyclization, methoxycarbonylation (Figure 7), polymerization (Figure 8), and isomerization, further demonstrating its versatility and industrial potential. As seen in Table 6, for the cyclization of 1,5-dienes, a combination of RuO_2_-H_2_O and the strong oxidant NaIO_4_ was employed as the catalyst. The reaction occurred at a temperature of 25 °C and was completed within a few minutes, resulting in the formation of cis-tetrahydrofuran derivatives with a selectivity ranging from 37% to 50%. However, side reactions such as the oligomerization/polymerization of olefins, isomerization of double bonds, premature termination, or competing intermolecular coupling can significantly impact yield and selectivity, especially under conditions favoring intermolecular processes or when using reactive catalysts [106]. The methoxycarbonylation of 1-hexenyl is performed using water-soluble palladium(II) complexes with phenoxy-type ligands. This reaction occurs at a temperature of 90 °C under a carbon monoxide pressure of 59.2 atm and lasts for 20 h. The process results in a 92% conversion rate and a 92% yield of the ester. Palladium is an effective catalyst for reactions that introduce carboxyl groups via carbon monoxide [107].

Additionally, the polymerization of ethylene utilizes a nickel(II) complex with a P^O ligand. This process is carried out at temperatures ranging from 50 to 70 °C and at a pressure of 39.5 atm, leading to the production of semi-crystalline linear polyethylene [108].

The isomerization of C_4_ olefins to butenes is performed using a nickel(0)-TPPTS-cyanide complex in the presence of NaBH_4_. This reaction takes place over a temperature range of 0 to 20 °C and at a pH of 9.5. It demonstrates a high conversion rate (TOF 3600 h^−1^), highlighting the excellent performance of the catalytic system [109]. The reactions summarized in the table highlight the significance of aqueous two-phase catalysis in various organic transformations, demonstrating high selectivity, efficiency, and the potential for catalyst recycling. Among all the reactions, hydroformylation stands out, as the chains and alcohols produced from this process serve as raw materials for nearly all chemical industries. Furthermore, each reaction not only yields valuable products but also contributes to important advancements in the field of biphasic catalysis in aqueous media, marking it as a pioneering and environmentally friendly approach.

## 4. Conclusions

Aqueous biphasic catalysis has emerged as a sustainable and efficient method for various organic transformations, providing a greener alternative to traditional solvent-based systems. This approach has attracted significant interest in both academic and industrial research due to its unique advantages, such as improved catalyst recyclability, a reduced environmental impact, and selective phase separation. By using water as the primary solvent, it minimizes the reliance on volatile organic compounds, aligning well with green chemistry principles. Transition metal catalysts, including those made from palladium, rhodium, cobalt, and nickel, are central to these systems, facilitating a wide array of reactions with high conversion rates and selectivity. The design of water-soluble ligands, such as hydrophilic phosphines and various others, is crucial for optimizing catalyst performance, stability, and phase compatibility. Additionally, carefully controlled reaction conditions, such as temperature, pressure, and pH, are essential for maintaining efficiency and prolonging the catalyst lifespan. Despite these advantages, challenges like limited substrate solubility and catalyst leaching remain. This has prompted ongoing research into better ligand architectures and phase-transfer strategies. Addressing these limitations through innovations in catalyst immobilization, continuous flow processes, and expanding substrate scope is vital to unlocking the full potential of aqueous biphasic catalysis. As research in this field advances, its integration into large-scale industrial applications is expected to increase, further solidifying its role in the future of sustainable chemical synthesis.

## Figures and Tables

**Table 1:** Aqueous biphasic Wacker oxidation of olefins.

Substrate	Products	Catalyst	Conditions	Additives	Catalytic Activity	References
C_12_–C_20_ olefins	2-methyl ketones	PdCl_2_	**Oxidant**: tert-butyl-hydroperoxide **Temperature**: 80 °C **Time**: 2–7 h **Stirring**: 300 rpm	n-paraffins	90–95% (Conversion)	[9]
Terminal C_6_ Olefins	2-alkanones	PhenS*Pd(OAc)_2_	**Oxidant**: O_2_, **Temperature**: 100 °C **Pressure**: 30 bar	NaOAc, NaOH	>99% (Selectivity)**,** 99% (Yield)	[10]
C_8_ olefins	cyclooctene oxide and styrene oxidants	Mn(salen)-Chit	**Oxidants**: m-CPBA, t-BuOOH, H_2_O_2_ **Temperature**: 25 °C **Time**: 24 h	-	3–39% (Yield), 12–160 h^−1^ (TOF)	[11]

**Notes:** C_12_–C_20_ olefins: n-paraffins reduced isomerization and improved selectivity; terminal C_6_ Olefins: catalysts were recyclable for multiple cycles; C_8_ olefins: the immobilized catalyst is more active and selective than the homogeneous catalyst.

**Table 2:** The aqueous biphasic hydrogenation of olefins.

Substrate	Products	Catalyst	Conditions	Additives	Catalytic Activity	References
C_4_–C_8_ olefins	C_4_–C_8_ alkanes, and alcohols derived from hydroformylated olefins	HRh(CO)(TPPMS)_3_, Rh(μ-Pz)(CO)(TPPMS)]_2_, RuCl_2_(TPPTS)_3_, RhH(CO)(TPPTS)_3_	**Temperature:** 70–150 °C **Time:** 1–24 h **Pressure:** 9.8–98.7 atm H_2_	ZnCl_2_, NaCl	18.81–100% (Conversion), 91–100% (Selectivity), 78–95% (Yield).	[12,13,14,15]
C_5_–C_10_ olefins	C_5_–C_10_ alkanes, alcohols derived from hydroformylated olefins and ethylbenzene derived from styrene	Raney Ni, [Rh(COD)Cl]_2_, palladium nanoparticles (immobilized within the walls of hollow polymeric microspheres), RuCl_2_(TPPMS)_3_(DMSO), PVP stabilized Rh nanoparticles	**Temperature:** 25–120 °C **Time:** 1–22 h **Pressure:** 0.99–34 atm H_2_ **Stirring:** 1600–2000 rpm	Catalytic nanoreactors (TPP@CCM1 or TPP@CCM2), electrolytes	34–100% (Conversion), 86–95% (Selectivity), 90–99% (Yield)	[24,25,26,27,28]
C_6_–C_12_ olefins	C_6_–C_12_ alcohols derived from hydroformylated olefins	Co/Ph_2_P(CH_2_CH_2_O)nCH_3_, Co/nBuPhP(CH_2_CH_2_O)nCH_3_, Ru/PTA, Pt/PTA, Rh nanoparticles/Ph_2_P(CH_2_CH_2_O)22CH_3_, [η^6^-(2-phenoxyethanol)RuCl(NH)]Cl, [η^6^-(2-phenoxyethanol)RuCl(S)]Cl, [η^6^-(2-phenoxyethanol)RuCl(O)]Cl, Ru(CO)_3_(TPPMS)_2_ (I), RuH_2_(CO)(TPPMS)_3_ (II)	**Temperature:** 20–100 °C **Time:** 1–8 h **Pressure:** 1–27.6 atm H_2_ **Stirring:** 500–630 rpm	-	41–100% (Conversion), 92–100% (Selectivity)	[16,17,18,19,29]
C_7_–C_18_ olefins	C_7_–C_18_ alkenes	Rh nanoparticles/Ph_2_P(CH_2_CH_2_O)_16_CH_3_ (thermoregulated)	**Temperature:** 60 °C **Time:** 1–2 h **Pressure:** 9.8 atm H_2_	-	87–100% (Conversion), 500–2000 h^−1^ (TOF)	[20]
Alfa-pinene	Cis and trans pinane	Rh nanoparticles/PVA, Ru nanoparticles stabilized by ammonium surfacants	**Temperature:** 25–70 °C **Time:** 1–3 h **Pressure:** 9.8–19.7 atm H_2_	-	96–99.9% (Conversion), 98.9–99% (Selectivity)	[21,22]
Polybutadiene	Hydrogenated polybutadiene	Rh/TPPTS	**Temperature:** 100 °C **Time:** 20–30 min **Pressure:** 19.7 atm H_2_ **pH:** 7	Cationic DTAC and Brij-35	255–1245 h^−1^ (TOF)	[23]

**Notes:** C_4_–C_8_ olefins: HRh(CO)(TPPMS)_3_ demonstrated resistance to poisoning by dibenzothiophene (30 ppm), indicating potential applicability in sulfur-rich systems such as naphtha cuts. The addition of salts (e.g., NaCl) improves hydrogenation rates by increasing aldehyde coordination to the catalyst; C_5_–C_10_ olefins: Activated Pd-EPS exhibited superior reusability due to enhanced activity. The microspheres encapsulate and concentrate hydrophobic olefins within their hydrophobic cavity in the aqueous phase, promoting efficient reaction without the need for co-solvents or surfactants. The microenviroment provided by the microspheres also minimizes side reactions. Electrolytes such as NaCl or Na_2_SO_4_ improved catalytic activity by affecting the micellar interphase. In contrast, K⁺ salts (e.g., KCl or K_2_SO_4_) slowed the reaction. Adding excess TPPMS or DMSO ligands reduced the hydrogenation rate by stabilizing the catalytic precursor and decreasing active site availability. Polybutadiene: TOF decreases under acidic or basic conditions.

**Table 3:** The aqueous biphasic hydroformylation of olefins to the same carbon atoms produced aldehydes.

Substrate	Catalyst	Conditions	Additives	Catalytic Activity	References
C_2_–C_4_ olefins	Rh/TPPTS, HRh(CO)(TPPTS)_3_	**Temperature:** 65–130 °C **Pressure:** 14.8–49.3 atm syngas **pH:** 5–6	-	92.5–99% (Selectivity)	[30,31]
C_3_–C_7_ olefins	RhH(CO)(TPPTS)_3_, [Rh(m-Pz)(CO)(TPPTS)]_2_, trans-Mo(CO)_4_(p-PySO_3_Na)_2_, RhCl(CO)(TPPMS)_2_, RhCl(CO)(TPPDS)_2_, RhCl(CO)(TPPTS)_2_, [HRu(CO)(CH_3_CN)(TPPTS)_3_]BF_4_	**Temperature:** 50–150 °C **Time**: 3–72 h **Pressure:** 13.8–98.7 atm syngas **Stirring:** 760–1200 rpm	-	40–100% (Conversion), 8–50% (Selectivity), 3–95% (Yield)	[32,33,34,35,95]
C_5_–C_12_	RhH(CO)(TPPTS)_3_, rhodium polyethylene glycolate, (RhH(CO)(TPPTS)_3_, Rh/PETPP, RhH(CO)(TPPTS)_3_	**Temperature:** 40–130 °C **Time:** 0.66–200 h **Pressure:** 39.5–118.4 atm syngas **Stirring:** 600–760 rpm	Monoethanolamine (MEA)	17–99% (Conversion), 98% (Selectivity), 72–95.5% (Yield)	[36,37,38,39,40]
C_6_ olefins	CoCl_2_(TPPTS)_2_, RhH(CO)(TPPTS)_3_, RhH(CO)(TPPTS)_3_, H_2_Ru(CO)(TPPMS)_3_, [Rh(CO)(μ-Pz)(TPPTS)]_2_	**Temperature:** 69.8–100 °C **Time:** 3–26 h **Pressure:** 17.2–88.8 atm syngas **Stirring:** 600–760 rpm	-	87–100% (Conversion), 68% (Selectivity), 90% (Yield)	[41,42,43,89]
C_6_–C_12_ olefins	Ph_2_P(CH_2_CH_2_O)_16_CH_3_, HRh(CO)(TPPTS)_3_, RhCl(CO)(TPPTS)_2_ modified with TPPDS, Rh(acac)(CO)_2_ with TPPTS/TPPDS/CDPPDS, TPPTS-Rh/SiO_2_, [Rh(acac)(CO)_2_] and TPPTS, Rh(acac)(CO)_2_ and water-soluble phosphine ligands, [RhH(CO)(TPPTS)_3_], [Rh(μ-Pz)(CO)(m-TPPTS)]_2_	**Temperature:** 70–120 °C **Time:** 0.5–200 h **Pressure:** 9.9–69.1 atm syngas **Stirring:** 760–1200 rpm	CTAB, [OctMim]Br	24.3–99.5% (Conversion), 57.9–95.7% (Selectivity), 93–97% (Yield), 8.95–11.88 (TON), 10–1614 h^−1^ (TOF)	[3,6,44,45,46,47,48,49]
C_8_ olefins	[Rh(sulphsal-X-R)(COD)], CoCl_2_(BiphTS)_2_, [RhCl(COD)]_2_, Rh(acac)(CO)_2_/TPPTS, mononuclear Rh(I)-salicylaldimine complex (9), trinuclear Rh(I)-salicylaldimine complex (10), rhodium complex ([Rh(acac)(CO)2]) embedded in phosphine-functionalized amphiphilic nanogels (TPP@NG), [Rh(acac)(CO)_2_] coordinated to BMOPPP ligands within the hydrophobic core of CCM, BMOPPP-functionalized micelles synthesized via RAFT polymerization, rhodium(I)-based mono-, di-, and trinuclear PTA complexes, CO-modified analogs, [Rh(cod)Cl]_2_/TPPTS, Rh/TPPTS, [Rh(cod)Cl]_2_/TPPTS, Ph_2_P(CH_2_CH_2_O)ₙMe, [Rh(acac)(CO)_2_]/TPPTS, Rh-nixantphos@CCM, [Rh(cod)Cl]2/TPPTS	**Temperature:** 75–180 °C **Time:** 3–20 h **Pressure:** 19.7–88.8 atm syngas **Stirring:** 300–2750 rpm **pH:** 5.5	CTAB, RAME-β-CD, AC-WV, cyclodextrins, nonionic latex, anionic latex (sodium 4-vinylbenzylsulfonate), cationic latex (4-vinylbenzyltrimethylammonium tetrafluoroborate)	98–99% (Conversion), 49–99% (Selectivity), 8–98.5% (Yield), 365 (TON), 4.6–742 h^−1^ (TOF)	[50,51,52,53,54,55,56,57,58,59,60,61,62,63,64,90]
C_8_–C_14_ olefins	CoCl_2_(TPPTS)_2_, Rh/Ph_2_P-(CH_2_)_10_-PO_3_Na_2_, Rh/Ph_2_P-(CH_2_)_12_-PO_3_Na_2_), Rh/TPPTS, HRh(CO)(TPPTS)_3_, [Rh(acac)(CO)_2_] combined with SulfoXantPhos, CoCl_2_(TPPTS)_2_, Rh(acac)(CO)_2_/TPPTS	**Temperature:** 80–140 °C **Time:** 2–10 h **Pressure:** 19.7–78.9 atm syngas **Stirring:** 600–100 rpm	CTAB, Lutensol^®^ ON 70 (C10E7, non-ionic amphiphile), nonionic surfacants (Marlophen NP 9), Polymer latices, RAME-β-CD	75–98% (Conversion), 40–98% (Selectivity), 71.6–88% (Yield), 65–5046 h^−1^ (TOF)	[65,66,67,68,69,91,92]
C_10_ olefins	Rh/β-cyclodextrin-based phosphane ligand, PEO–DPPPA/Rh, Rh(acac)(CO)_2_/M1NPS, Rh(acac)(CO)_2_/D2NPS, Rh(CO)_2_(acac)/2,7-bis(SO_3_Na)-xantphos, [RhH(CO)(TPPTS)_2_]^6−^, Rh/TPPTS, Rh/sulfoxantphos, [Rh(acac)(CO)_2_]/TPPTS	**Temperature:** 76.8–130 °C **Time:** 3–240 h **Pressure:** 40.8–50 atm syngas **Stirring:** 500–1500 rpm	RAME-β-CD, 2,6-dimethyl-β-CD	99.5–100% (Conversion), 93–97% (Selectivity), 39–99% (Yield)	[70,71,72,73,74,75,76]
C_10_–C_18_ olefins	Rh(CO)_2_(acac)/TPPTS, Rh/Ph2P(CH2CH2O)_22_CH_3_, RhCl(CO)(TPPTS)_2_, Rh(acac)(CO)_2_/TPPTS, RhCl(CO)(TPPTS)_2_, Rh(acac)(CO)_2_/1-(4-tert-butylbenzyl)-1-azonia-3,5-diaza-7-phosphaadamantyl bromide	**Temperature:** 80–120 °C **Time:** 1–6 h **Pressure:** 19.7–49.3 atm syngas **Stirring:** 400–1500 rpm	PEG-substituded pillar[5]arene, DLCS, OS-CDs, β-CD-(OSG–Me)_1_, cationic gemini and trimeric surfactants, RAME-β-CD, native β-CD	72–100% (Conversion), 51–95% (Selectivity), 94% (Yield), 157–1111 h^−1^ (TOF)	[77,78,79,80,81,82,83]
C_12_ olefins	RhCl(CO)(TPPTS)_2_, RhCl(CO)(2-MOTPPTS)_2_, RhCl(CO)(4-MOTPPTS)_2_, [Rh(acac)(CO)_2_], Rh/TPPTS, RhCl(CO)(TPPTS)_2_	**Temperature:** 80–130 °C **Time:** 2–6 h **Pressure:** 9.9–49.3 atm syngas **Stirring:** 0–1000 rpm	CTAB, cyclodextrins, RAME-β-CD, Gemini surfactants (cationic) with varying spacers, CPB, SDS, DBS, Triton X-100, Brij 35, 1-pentanol, 1-heptanol	8–94% (Conversion), 81.8–90% (Selectivity), 94% (Yield), 883–1200 h^−1^ (TOF)	[1,84,85,86,87,88]

**Notes:** C_2_–C_4_ olefins: A pH level of 5–6 is optimized to suppress side reactions such as aldol condensation. Auxiliary agents, like polyethylene glycol, can improve reaction rates by increasing propene solubility in water. C_3_–C_7_ olefins: The active catalyst deactivates above 130 °C. Selectivity to oxygenated products improves at higher CO:H_2_ ratios. C_5_–C_12_: The rhodium catalyst showed high stability, retaining activity for up to 200 h without significant leaching (<5 ppm Rh in the organic phase). Achieved 70% total olefin conversion in 42 h with MEA, compared to 17% in the same timeframe without MEA. C_6_ olefins: Lower temperatures (e.g., 60 °C) favor aldehyde formation (up to 83% selectivity). Higher temperatures (e.g., 120 °C) increase isomerization and reduce aldehyde yield. C_6_–C_12_ olefins: The synergistic effect of TPPTS and TPPDS significantly enhances regioselectivity toward linear aldehydes. Lower pressures favor linear aldehyde formation over branched products. CDPPDS significantly increases the reaction rate but slightly reduces the normal aldehyde selectivity compared to TPPTS. The high surface area of SiO_2_ enhances catalytic activity by providing space for the hydroformylation reaction. Additives like [OctMim]Br enhance substrate solubility in water and improve catalyst–substrate interactions by forming emulsions. They also allow for clear phase separation after the reaction, enabling easy catalyst recovery. C_8_ olefins: Higher temperatures favored branched aldehyde formation due to substrate isomerization. Mononuclear complexes are less active than di- and trinuclear complexes. CO-modified catalysts exhibit better regioselectivity but lower activity compared to COD-based analogs. C_8_–C_14_ olefins: TPPTS facilitates the separation of organic and aqueous layers, while CTAB increases the interfacial area for improved reaction rates. C_10_ olefins: The PEO–DPPPA/Rh catalyst utilizes a thermoregulated phase-transfer process. It remains in the aqueous phase at lower temperatures and transitions to the organic phase at higher temperatures (above the cloud point, Cp = 92 °C). It also retains its high efficiency (conversion >94.0% and yield >94.0%) even after 20 recycling cycles. RAME-β-CD enhances the reaction by forming inclusion complexes, improving mass transfer at the aqueous–organic interface. The degree of methylation on RAME-β-CD significantly affects catalytic activity, with higher methylation improving substrate accessibility at the interface. C_10_–C_18_ olefins: Without stirring, conversion rates decrease, and hydrogenation and isomerization by-products increase, due to the lower CO transfer. RAME-β-CD increases conversion at higher temperatures by promoting mass transfer and enhancing interfacial activity. At lower temperatures (80 °C), RAME-β-CD inhibits conversion due to the strong inclusion of the ligand in the cyclodextrin cavity. C_12_ olefins: Gemini surfactants facilitated higher conversions and selectivity due to their ability to lower surface tension and form compact micellar structures.

**Table 4:** Aqueous biphasic epoxidation of olefins.

Substrate	Catalyst	Conditions	Additives	Catalytic Activity	References
C_3_–C_6_ olefins	[p-C_5_H_5_NC_16_H_33_]_3_[PW_4_O_16_], [C_5_H_5_N(CH2)_15_CH_3_]_3_[PW_4_O_16_], Mn(TDCPP)Cl and Iron porphyrins	**Oxidant:** H_2_O_2_ **Temperature:** 25–65 °C **Time:** 1–4 h	Hexafluoroacetone hydrate (HFAH)	90–99% (Conversion), 99.5% (Selectivity)	[96,97,98]
C_6_–C_8_ olefins	Polyoxometalate (POM) derivatives	**Oxidant:** H_2_O_2_ **Temperature:** 70 °C **Time:** 4 h	-	95% (Conversion), 87–95% (Selectivity)	[99]
Terpenes (Limonene + alfa-pinene)	Dimethyldioxirane (DMDO) generated in situ from oxone (potassium peroxymonosulfate) and acetone	**Oxidant:** Oxone **Temperature:** 25 °C **Time**: 45–90 min	-	100% (Conversion), 99–100% (Yield)	[100]

**Notes:** C_3_–C_6_ olefins: The use of hexafluoroacetone hydrate suppresses side reactions, stabilizes the catalyst, and enhances the selectivity and efficiency of the oxidation process.

**Table 5:** Aqueous biphasic hydroaminomethylation of olefins.

Substrate	Catalyst	Conditions	Additives	Catalytic Activity	References
C_6_–C_12_ olefins	Rh nanoparticles/Ph_2_P(CH_2_CH_2_O)_16_CH_3_, [Rh(cod)Cl]_2_ combined with Na-TPPTS, RhCl(CO)(TPPTS)_2_, [Rh(cod)Cl]_2_/Sulfoxantphos, RhCl(CO)(TPPTS)_2_	**Temperature:** 100–130 °C **Time:** 4–6 h **Pressure:** 29.6–59.2 atm syngas or CO_2_ or CO:H_2_	Morpholine salts, CTAB	80.1–99% (Conversion), 51.4–98% (Selectivity)	[101,102,103,104,105]
C_10_–C_16_ olefins	Rh/TPPTS, Rh/Sulfoxantphos, Rh(acac)(CO)_2_, combined with the ligand SulfoXantphos	**Temperature:** 80–125 °C **Time:** 30 h **Pressure:** 29.6–49.3 atm syngas	RAME-β-CD	80% (Selectivity)	[93,94]

**Notes:** C_6_–C_12_ olefins: CTAB significantly accelerates the reaction by increasing the interfacial area and enriching rhodium species at the interface. Higher temperatures reduce selectivity for tertiary amines and favor side reactions. Increased pressure enhances conversion but reduces selectivity for linear amines.

**Table 6:** Other reactions conducted using aqueous biphasic catalytic conversion of olefins.

Substrate	Reaction	Products	Catalyst	Conditions	Additives	Catalytic Activity	References
1,5-dienes	Cyclization	cis-Tetrahydrofuran derivatives	RuO_2_·_2_H_2_O	**Oxidant:** NaIO_4_ **Temperature:** 25 °C **Time:** Few min	-	37–50% (Selectivity)	[106]
1-hexene	Methoxycarbonylation	Esters	Water-soluble palladium(II) complexes with phenoxyimine ligands	**Temperature:** 90 °C **Time:** 20 h **Pressure:** 59.2 atm CO	-	92% (Conversion), 92% (Yield)	[107]
Ethylene	Polymerization	Linear semicrystalline polyethylene	P∧O-chelated nickel(II) complex	**Temperature:** 50–70 °C **Pressure:** 39.5 atm	-	**-**	[108]
C_4_ olefins	Isomerization	Butenes	Nickel(0)–TPPTS–cyanide complex	**Temperature:** 0–20 °C **Time:** 15 min to 1 h **pH:** 9.5	NaBH_4_	3600 h^−1^ (TOF)	[109]

## Data Availability

Data are contained within the article.
